# Sex Differences in Substance Use, Prevalence, Pharmacological Therapy, and Mental Health in Adolescents with Attention-Deficit/Hyperactivity Disorder (ADHD)

**DOI:** 10.3390/brainsci12050590

**Published:** 2022-05-02

**Authors:** Francisca Castellano-García, Ana Benito, Antonio Jovani, Alejandro Fuertes-Sáiz, María Isabel Marí-Sanmillán, Gonzalo Haro

**Affiliations:** 1 TXP Research Group, Universidad Cardenal Herrera-CEU, CEU Universities, 12006 Castellon, Spain; francisca.castellanogarcia@uchceu.es (F.C.-G.); anabenitodel@hotmail.com (A.B.); antonio.jovani@hotmail.com (A.J.); alejandro.fuertessaiz@uchceu.es (A.F.-S.); maria.mari1@uchceu.es (M.I.M.-S.); 2 Department of Education Sciences, CEU Cardenal Herrera University, 12006 Castellon, Spain; 3 Torrente Mental Health Unit, Hospital General de Valencia, 46900 Torrente, Spain; 4 Department of Mental Health, Consorcio Hospitalario Provincial de Castellon, 12002 Castellon, Spain; 5 Psychiatry Service, Hospital La Salud Valencia, 46021 Valencia, Spain

**Keywords:** ADHD, sex differences, adolescence, substance use disorder, dual disorder

## Abstract

Sex differences are poorly studied within the field of mental health, even though there is evidence of disparities (with respect to brain anatomy, activation patterns, and neurochemistry, etc.) that can significantly influence the etiology and course of mental disorders. The objective of this work was to review sex differences in adolescents (aged 13–18 years) diagnosed with ADHD (according to the DSM-IV, DSM-IV-TR and DSM-5 criteria) in terms of substance use disorder (SUD), prevalence, pharmacological therapy and mental health. We searched three academic databases (*PubMed, Web of Science, and Scopus*) and performed a narrative review of a total of 21 articles. The main conclusions of this research were (1) girls with ADHD are more at risk of substance use than boys, although there was no consensus on the prevalence of dual disorders; (2) girls are less frequently treated because of underdiagnosis and because they are more often inattentive and thereby show less disruptive behavior; (3) together with increased impairment in cognitive and executive functioning in girls, the aforementioned could be related to greater substance use and poorer functioning, especially in terms of more self-injurious behavior; and (4) early diagnosis and treatment of ADHD, especially in adolescent girls, is essential to prevent early substance use, the development of SUD, and suicidal behavior.

## 1. Introduction

Sex differences in patients with mental disorders have been little studied [1,2], with the few studies on the subject being limited to analyzing quantitative differences between the sexes [3]. These differences can significantly influence the etiology and course of mental disorders. The literature published in recent years in relation to sex differences indicates that these dichotomies begin to manifest in pre- and early postnatal development. The hypothalamic–pituitary–gonadal (HPG) axis is thought to be responsible for this differentiation through the production of high levels of gonadal steroid hormones. [4]. For example, testosterone produces masculinization and defeminization in neural circuits in males, and the absence of testosterone produces a female neural phenotype [5]. After the first year of postnatal life, the HPG axis remains inactive until the onset of puberty [4]. At around 9 years of age in girls and one year later in boys, the hypothalamic–pituitary–adrenal axis is activated. This causes the increased production of adrenal androgens, thus initiating the development of secondary sexual characteristics, including the growth of pubic or axillary hair [6].

During puberty, there is an increase in sex hormones which results in changes in the activation and organization of the brain, leading to significant changes in its structure [7]. One of the best known and most replicated sex differences in brain development studies relates to overall volume (brain and intracranial), which is increased throughout development in boys compared to girls [8]. Grey matter volumes (cortical and subcortical) also show an inverted U-shaped growth trajectory with a maximum peak in girls at 8.5 years of age and approximately two years later in boys [9]. These findings have led to growing interest and an increase in studies in adolescents [8]. In fact, the study of sex differences may provide relevant information to explain the etiology of several diseases with an onset before or during adolescence and significant differences in prevalence according to sex [10].

One of the most worrying issues during adolescence is substance use. Epidemiological studies indicate that early adolescent use of alcohol, marijuana, and cocaine increases the risk of substance use disorder (SUD) in adulthood [11]. Indeed, risk taking and subsequent substance use during this developmental period increases the likelihood of developing SUD more so in boys than in girls [12]. Predisposing risk factors, such as impulsiveness [13], novelty seeking [14], exposure to early adversity [15], and other pre-existing conditions including attention deficit hyperactivity disorder (ADHD) [16], can also lead to the early use of substances.

ADHD affects 5–10% of the child population [17,18,19]. Although the condition was initially thought to decline or disappear during adulthood, research over the last three decades has found that it persists into adolescence in 50–60% of cases [20,21,22] and may even last through the whole lifespan [23,24,25]. The main characteristic of ADHD is the presence of a persistent pattern of inattention and/or hyperactivity–impulsivity that interferes with the functioning or general development of people with the disorder [26]. ADHD is classified into three subtypes according to whether there is a predominance of (1) combined presentation; (2) predominantly inattentive presentation; or (3) predominantly hyperactive/impulsive presentation. The core symptoms occur in childhood and early adolescence (before the age of 12) although their expression changes during adolescence with the motor hyperactivity being mitigated but the impulsivity and attention deficit persisting [27].

ADHD frequently co-occurs with other psychiatric disorders, such as conduct disorder, obsessive–compulsive disorder, or SUD [26]. Childhood ADHD predicts greater initial exposure to substances from earlier ages and a more rapid progression of substance use during adolescence [28] and in young adults [29]. This means that the disorder itself is considered a risk factor for SUD [30,31]. Follow-up studies have highlighted a higher prevalence of substance use in the population of adolescents with ADHD relative to the general population [32,33,34]. Nonetheless, a review on sex differences in relation to the disorder has never been published. In this context, we believe that a review focusing on ADHD in adolescence was required given that sex differences are more evident during this period, and it also often coincides with the start of substance use. Thus, the objective of this study was to conduct a narrative review of the academic literature on sex differences in substance use (if the subject uses the substance), substance use disorder (if the subject is diagnosed with a substance use disorder, including both abuse (the substance is consumed despite the problems and negative consequences it causes) and dependence (substance use causing tolerance, withdrawal, and/or pattern of compulsive use)), prevalence, pharmacological therapy, and mental health in adolescents with ADHD (understanding sex as a biological variable [3] operationalized as sex assigned at birth: male or female). Our secondary objective was to review sex differences in variables related to substance use and SUD.

## 2. Materials and Methods

### 2.1. Search Strategy

The search strategy of this narrative review has taken into account the PRISMA checklist (except items 12–15 and 19–22) [35]. The protocol was registered with the Prospero Centre for Reviews and Dissemination on 19 February 2022 (CRD42022304765), available at the following address: https://www.crd.york.ac.uk/prospero/display_record.php?RecordID=304765 (accessed on 10 March 2022). The search for relevant studies was conducted in the PubMed (28 October 2021), Web of Science (12 November 2021), and Scopus (20 November 2021) databases. We used several combinations of keywords as follows: (1) ((ADHD OR Gender differences OR Sex differences) AND (Adolescents OR Teenagers OR Teens) AND (Dual diagnosis OR Dual diagnoses)AND (Substance use OR Substance addiction)); (2) ((ADHD) AND (Gender differences OR Sex differences) AND (Substance use OR Substance addiction) AND (Adolescents OR Teenagers OR Teens)); and (3) ((ADHD) AND (Gender differences OR Sex differences) AND (Adolescents OR Teenagers OR Teens)).

### 2.2. Inclusion and Exclusion Criteria

The inclusion criteria were: (1) a diagnosis of ADHD using questionnaires or a clinical interviews according to the DSM-IV [36], DSM-IV-TR [37], and DSM-5 [26] criteria; (2) substance use disorder and/or related variables; (3) studies conducted in adolescents aged 13 to 18 years; (4) data segregated by sex provided in the study report (understanding sex as a biological variable [3] operationalized as sex assigned at birth: male or female); and (5) the sample cohorts must have comprised 100 or more participants. Relevant studies were included irrespective of the type of study, publication language, date of publication, or the nationality or race of the participants considered. The exclusion criteria was the diagnosis of psychiatric disorders other than ADHD or ADHD+SUD. Gray literature was not included.

### 2.3. Data Extraction

Two authors independently extracted the information from the articles included in this work by using a standardized form. Any disagreements were resolved though discussion with two of the other authors until a consensus was reached. The variables extracted from the studies were the names of the authors, publication year and country, population, sample age, cohort size, ADHD evaluation instruments used, sexual differences evaluated, substance use, study type, and study quality assessed using the Newcastle Ottawa Scale (NOS; [38]).

### 2.4. Quality Assessment

The NOS [38] was used to assess the methodological quality of the studies included in this review. This scale assigns a maximum of nine stars to the following domains: selection, comparability, exposure, and study results. The NOS has two formats depending on the design of the study to be assessed: case-control or cohort studies. We used the version corresponding to the design of each study included in this work. For comparative studies of differences between groups we have used the adaptation for cross-sectional studies by Modesti et al. [39].

## 3. Results

**Figure 1:**
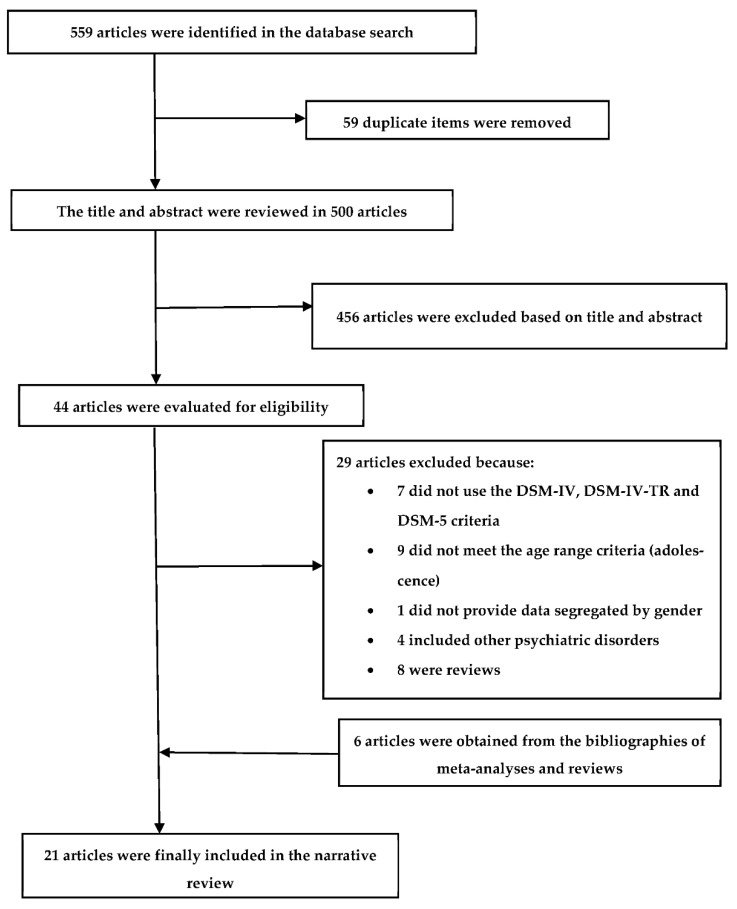
Flowchart of the article selection process.

The article selection process, which lasted two months, is summarized in Figure 1. The database search produced hits for 559 articles, of which 59 duplicates were eliminated, leaving 500 manuscripts. Another 456 articles were eliminated after selection based on the titles and abstracts. The full text was then examined in the remaining 44 articles. Of these, 29 were eliminated for the following reasons: 7 had not used the DSM-IV, DSM-IV-TR, and DSM-5 criteria; 9 did not meet the age range (adolescence) criteria; 1 had not provided data disaggregated by sex; 4 had included other psychiatric disorders; and 8 were reviews. Together with the remaining 15 articles, another 6 articles were identified and incorporated by searching the references used in the meta-analyses and reviews cited above, meaning that a total of 21 articles were finally included in this review [40,41,42,43,44,45,46,47,48,49,50,51,52,53,54,55,56,57,58,59,60]. The most significant characteristics from these studies [40,41,42,43,44,45,46,47,48,49,50,51,52,53,54,55,56,57,58,59,60] are presented in Table 1a,b.

The results of these studies were summarized as sex differences in substance use and sex differences in variables that may be related to substance use and addiction: the prevalence of ADHD and pharmacological therapy, cognitive and academic functioning, and other variables. Table 2 shows the results referring to sex differences in the use of substances. Table 3 shows the results referring to sex differences in the prevalence of ADHD and pharmacological therapy. Table 4 shows the results referring to sexual differences in cognitive and academic functioning. Finally, Table 5 shows the results referring to other sexual differences.

## 4. Discussion

Our review synthesizes the literature on sex differences in substance use, substance use disorder, prevalence, pharmacological therapy, and mental health in adolescents with ADHD. Our results show that ADHD was associated with SUD in adolescence and that girls with ADHD were at an increased risk for some types of SUD, including tobacco, alcohol, marijuana, and cannabis [43,44,45,46,52,55,59]. Indeed, the body of research suggesting that girls with ADHD may be at increased risk for SUD is growing [29,32,34,61,62,63,64]. Two studies, by Biederman et al. (2002) [65] and Biederman and Faraone (2004) [66], indicated that ADHD in girls was a greater risk factor for SUD than ADHD in boys, noting that girls with the disorder were at particular risk in early adolescence. Along the same lines, Ottosen et al. (2019) [67] confirmed that girls with ADHD were at an increased risk of SUD, indicating that delayed diagnosis and the late initiation of treatment was a risk factor for the development of SUD. In contrast, other studies emphasized that the association between ADHD and SUD is greater in adolescent boys [51] and considered male sex to be a risk factor for this comorbidity [33,60,66].

Yildiz et al. (2020) [60] found that alcohol consumption in adolescents with ADHD was associated with a SUD comorbidity in boys and with hyperactivity–impulsivity in girls [45], with girls presenting greater SUD [55]. However, the two studies with participants with severe childhood ADHD, found no sex differences: both sexes started drinking alcohol earlier, and the consumption was more frequent than in individuals without the disorder [45,59]. This could be because the severity of ADHD in childhood is related to the risk of consumption or because sex does not modulate this relationship. This finding suggests that it is important to consider the severity of ADHD when evaluating sex differences.

Nevertheless, the results of the study by Madsen and Dalsgaard (2013) [53] were opposite to all the other work in the field, showing that the boys in the control group had a pattern of more intense and frequent alcohol consumption than those who followed pharmacological treatments for ADHD. However, the latter consumed more tobacco than the control group of boys. Moreover, they found no difference in alcohol and tobacco use between adolescent girls with and without ADHD. These results could be justified, on the one hand, precisely by the fact that diagnosis and pharmacological intervention tends to be earlier in boys than in girls, thus facilitating improvement of the symptoms of the disorder in boys rather than girls. On the other hand, published studies addressing drug therapy indicate that people with ADHD who receive prompt treatment show the same rates of SUD as age-matched community controls [16,68]. In other words, when started early, medication lowers the risk of substance use [69], and this lower risk is sustained in the long term among men [70]. This seems to indicate the importance of early diagnosis and treatment, especially in girls.

Our study also reviewed sex differences in other variables related to substance use and SUD in adolescents with ADHD. Two of the related variables are the prevalence of ADHD and drug therapy. Our results indicated that boys with ADHD had more accentuated symptoms, and therefore, the prevalence was higher among them. We believe this would lead to an earlier diagnosis in boys than in girls [41,50,54], who are usually diagnosed later and have a significantly lower probability of receiving pharmacotherapy than boys [40,49]. Previous studies have already indicated that ADHD seems to affect more men than women, with the exact proportion differing between clinical and general population samples [37,71,72,73,74,75]. The lack of externalizing symptoms in girls and women with ADHD hampers both their referral and earlier diagnosis [75]. Furthermore, the inattentive-type ADHD, which usually involves less disruptive behaviors, is more often diagnosed in women, both in childhood and in adulthood [19]. This could perhaps contribute to the explanation of why girls with ADHD tend to be underdiagnosed [76,77], probably due to sex differences in the presentation of the symptoms and comorbidities of the disorder [65,72].

Indeed, the study of ADHD has long focused primarily on boys [78] despite the still relatively high percentage of girls with the disorder [79]. The samples in most published work comprised mainly men with only a few female participants. This situation has caused many girls to receive a late diagnosis, and even more worryingly, these patients often do not receive appropriate pharmacological treatment after their diagnosis [80,81]. Follow-up studies have further suggested that women with ADHD may have more serious problems than their male counterparts [67]. For example, they have a higher risk of substance use, admission to inpatient psychiatric units, and higher mortality rates than men [32,76,80]. These results support the idea that the current diagnostic criteria may not adequately detect girls with ADHD [82,83]. Most of these criteria refer to behaviors that are more easily manifested and observed in boys. Therefore, the DSM-5 continues to present a considerable limitation in this sense because the symptoms it lists for ADHD are not modulated by sex differences. This situation causes a significant obstacle when it comes to recognizing and diagnosing girls with ADHD, thus producing erroneous or late diagnoses, which can lead to a worsening of symptoms in this population over time [77].

Other variables analyzed in this review were sex differences in cognitive and academic functioning in adolescents with ADHD. We identified sex differences among individuals with ADHD in terms of cognitive functioning (especially executive functioning) and, within this, in impulsivity and attention deficit problems. Adolescent girls had higher levels of cognitive impulsiveness and motor impulsivity [47]; made more errors of omission (deficits in attentional control); had more deficits in design memory, general visual memory, verbal arithmetic skills, and working memory skills [56]; and had higher processing and encoding speed scores, greater inhibitory control, and lower vocabulary scores than adolescent boys with ADHD [57]. These findings are consistent with those obtained in two meta-analyses. The first, conducted by Hasson and Fine (2012) [84], found that boys with ADHD made more commission errors (inhibitory control) in the Conners’ Performance Test (CPT) than girls who, in turn, made more omission errors (attentional control). The second meta-analysis was carried out by Loyer Carbonneau et al. (2021) [78] and found significant differences between both sexes regarding inhibition capacity (motor response inhibition and interference control) and cognitive flexibility such that boys with ADHD presented more hyperactive behaviors and greater inhibition problems than girls with ADHD. Previous research has shown that girls with ADHD were more likely to have the inattentive subtype [19] and because of these problems in attentional control showed more difficulties in design memory, visual memory, perceptual and visuospatial reasoning, and working memory [56,65,72,85]. Consequently, it seems that neuropsychological functions are more affected in girls with ADHD compared to boys with the same disorder [58]. This finding coincides with previous studies indicating that ADHD affects neuropsychological functioning differently depending on sex [78,86,87]. The more deteriorated neuropsychological profile in girls might be related to the fact that girls tend to receive a later diagnosis and less frequently receive therapeutic interventions. In addition, cognitive and motor impulsiveness and deficits in cognitive and executive functioning may be related to the higher risk of substance use in girls. In turn, consumption could worsen these deficits and impulsiveness. Therefore, in the future, early diagnosis and intervention in girls with this disorder is of vital importance to help improve the symptoms of ADHD and prevent other problems associated with it (such as substance use) among women. We did not find any gender differences in adolescents with ADHD in relation to academic functioning. However, a review conducted in children showed that ADHD was frequently associated with poorer academic performance and that they were more likely to need to repeat a grade or even drop out of school [88]. In a similar vein, other studies have indicated that one of the factors that increases the risk of SUD in ADHD is academic failure [89]. That is, ADHD associated with a negative academic trajectory can lead to early substance use [90]. This points to the importance of specific programs to prevent school failure as well as substance use in adolescents with ADHD regardless of sex.

Importantly, in this review, we found that sex is related to suicidal behavior in adolescents with ADHD. Regarding girls, some studies linked the inattentive type of ADHD with an increased risk of suicidal behavior because inattention can be a particularly important deteriorating factor [91]. As we have seen, girls are usually diagnosed later [76,77], tend to have the inattentive type of ADHD [19], and are less likely to receive any type of treatment [40,49]. This often means that they present greater deterioration as a consequence of the difficulties and problems they suffered during childhood. In fact, follow-up studies have indicated that women with ADHD have more severe problems than men with the same disorder [32,76,80]. Another possible explanation for these differences in suicidal behaviors is that comorbidity of ADHD with substance use increases the risk of suicidal ideation and suicide attempts [92]. This could explain why girls with ADHD present more suicidal ideation because, as previously mentioned, they tend to present more SUD [60]. Nonetheless, we found a study that failed to identify any sex differences in terms of suicidal ideation and suicide planning, with both boys and girls with ADHD presenting the same risk [50]. This agrees with studies that found that the risk of alcohol consumption was similar in both sexes [45,59], perhaps indicating that the early deterioration in cases of severe childhood ADHD is so significant that it is not modulated by sex differences in adolescence. The study by Kessler et al. (2014) [50], which found that ADHD was associated with psychological problems, distress, and stress in both sexes, also concurs with previous reports of an absence of sex differences.

Finally, we must point out that our review has some limitations. The first is the age range we selected for the inclusion criteria (13 to 18 years). In most of the studies reviewed, the samples comprised children and adolescents, and the data obtained were not broken down into different age ranges to separate the childhood and adolescent periods. In other studies, the age range was much broader, encompassing childhood, adolescence, and young adulthood. However, this limitation can also be considered a strength because this is the period during which sex differences are most evident and substance use most often begins. Another relevant aspect to highlight is that most of the articles we reviewed had included more boys than girls, which could have biased their results. It is also worth noting the variability in these studies both in terms of the instruments used to assess ADHD and the variables of interest in each study. Another limitation was the small number of studies we found regarding each variable and the heterogeneity between them, which made it impossible to quantitatively synthesize the results through a meta-analysis. This meant that the PRISMA criteria [35] regarding quantitative synthesis of the results could not be met. Lastly, many of the studies we included were cross-sectional, meaning that we could not infer the direction of causality between ADHD and the variables studied.

## 5. Conclusions

In our review, we found sex differences in adolescents with a diagnosis of ADHD in terms of prevalence, pharmacological treatment, and mental health. There is evidence that girls with ADHD may be at greater risk of for some types of SUD (including tobacco, alcohol, marijuana, and cannabis) than boys with this disorder, especially in mild and moderate cases, where girls present more symptoms of hyperactivity and impulsivity. Meanwhile, adolescents of both sexes who had had severe childhood ADHD showed similar risk levels for substance use. Studies regarding the evolution of adolescents with ADHD from substance use to SUD (dual disorder) are scarce and did not show consistent sex differences. Boys seemed to have a higher prevalence of ADHD and more pronounced symptoms but were diagnosed earlier than girls with ADHD. In contrast, persistent ADHD was less frequent in adolescent girls (partly due to underdiagnosis), and they received treatment less frequently. This was likely both because of underdiagnosis and the fact that they more often presented the inattentive ADHD phenotype, which is associated with fewer disruptive behaviors. The delay in diagnosis, underdiagnosis, and lack of treatment, together with the greater deterioration in cognitive and executive functioning in girls, have all been proposed as reasons for the greater risk of SUD consequently leading to deterioration of functioning in girls with ADHD, especially leading them towards suicidal behavior. Thus, given all the above, early diagnosis and treatment of ADHD is essential, especially in adolescent girls, to help prevent early substance use, SUD, and suicidal behaviors.

## Figures and Tables

**Table 1:** (**a**)**.** Characteristics from sex differences’ studies on ADHD adolescents [40,41,42,43,44,45,46,47,48,49,50,51,52,53,54,55,56,57,58,59,60]. (**b**) Study type and quality evaluation from sex differences’ studies on ADHD adolescents [40,41,42,43,44,45,46,47,48,49,50,51,52,53,54,55,56,57,58,59,60].

(a)					
1st Author Year	Country	Population	Age	Sample	ADHD Assessment
Barbaresi et al., 2006	USA	School records and medical records (Rochester)	17 years	*N* = 379	DSM-IV
Byrd et al., 2013	USA	Data (NHANES)	12–15 years	*N* = 1906	DISC-IV (DSM-IV criteria)
Cole et al., 2008	USA	Sample obtained from clinics, pediatricians, schools, etc.	14 years	*N* = 268	Interview DICA-R/DICA-IV (DSM-III-R/DSM-IV criteria)
Disney et al., 1999	USA	Minnesota Twin Family Study	17-years	*N* = 1252	DICA-R (Clinical Interview, DSM-III-R)
Elkins et al., 2018a	USA	Minnesota Twin Family Study	14–18 years	*N* = 2510	DICA-R, SCID (DSM-IV, and DSM-III-R clinical interviews)
Elkins et al. 2018b	USA	Minnesota Twin Family Study participants	17 years	*N* = 3762	DICA–R (DSM-IV)
Elkins et al., 2020	USA	Minnesota Twin Study	14–17 years	*N* = 998	DICA–R (DSM-IV)DSM-5
Gökçe et al., 2017	Turkey	Hospital for Psychiatry and Neurology, Child and Adolescent Clinic	11–18 years	*N* = 156	SNAP-IV (DSM-IV criteria)
Hurtig et al., 2012	Finland	General population	16–18 years	*N* = 273	SWAN (DSM-IV-TR)
Huss et al., 2008	Germany	Children and adolescents in the KiGGS study	14–17 years	*N* = 236	Clinical interview (physician or professional psychologist)
Kessler et al., 2014	USA	The U.S. National Comorbidity Survey Replication Adolescent Supplement	13–17 years	*N* = 6483	Interview (CIDI) DSM-IV
Latimer et al., 2002	USA	Schools and clinics and mental health and justice centers	12–19 years	*N* = 135	DSM-IV
Lee et al., 2015	USA	National Longitudinal Study of Adolescent to Adult Health	13 years	*N* = 9719	DSM-IV
Madsen and Dalsgaard, 2013	Denmark	Psychiatric and Neurology Centre	13–18 years	*N* = 219	DAWBA (clinical interview)
Pineda et al., 1999	Colombia	Schools in Manizales	12–17 years	*N* = 177	DSM-IV
Regan and Tubman, 2020	USA	Adolescents with ADHD and outpatient treatment for substance use	12–18 years	*N* = 394	Entrevista clínica (UM-CIDI)
Rucklidge, 2006	New Zeland	Participants referred to a specialised psychiatric service	13–17 years	*N* = 114	DSM-IV-TR clinical interview
Rucklidge and Tannock, 2001	Canadá	Deparment of Psychiatry and Hospital for Sick Children	13–16 years	*N* = 107	Clinical interview (DSM-IV)
Seidman et al., 2005	USA	Data provided by two previous studies	13–17 years	*N* = 105	DSM-III, DSM-III-R, DSM-IV, andDSM-IV-TR
Selinus et al., 2016	Suecia	The Child and Adolescent Twin Study in Sweden (CATSS)	15 years	*N* = 506	DSM-IV
Yildiz et al., 2020	Turkey	Hospitalized adolescents (CEMATEM clinic)	Adolescents aged < 18 years	*N* = 105	DSM-IV
(**b**)					
**1st Author Year**		**Study Type**		**NOS Quality**	
Barbaresi et al., 2006		Cohort		8	
Byrd et al., 2013		Case-control		9	
Cole et al., 2008		Case-control		7	
Disney et al., 1999		Case-control		8	
Elkins et al., 2018a		Cohort		7	
Elkins et al., 2018b		Cohort		7	
Elkins et al., 2020		Case-control		9	
Gökçe et al., 2017		Comparative		5	
Hurtig et al., 2012		Case-control		8	
Huss et al., 2008		Case-control		6	
Kessler et al., 2014		Cohort		9	
Latimer et al., 2002		Comparative		5	
Lee et al., 2015		Cohort		6	
Madsen and Dalsgaard, 2013		Case-control		7	
Pineda et al.,1999		Comparative		8	
Regan and Tubman, 2020		Case-control		9	
Rucklidge, 2006		Case-control		7	
Rucklidge and Tannock, 2001		Case-control		7	
Seidman et al., 2005		Case-control		8	
Selinus et al., 2016		Cohort		8	
Yildiz et al., 2020		Comparative		4	

**Table 2:** Sex differences in substance use and substance use disorder.

Substance Use	Girls/Boys	No Sex Differences
Nicotine	Adolescent girls with ADHD patients suffered more frequently nicotine use disorder [43,44], with a probability of nicotine use up to 5 times higher than adolescent boys with ADHD [46]. However, white males smoked more throughout adolescence [52].	
Alcohol	Adolescent boys with ADHD and SUD consumed more alcohol, with male sex being a risk factor for these behaviors [60]. However, adolescent girls with more hyperactive–impulsive symptoms consumed more alcohol [45] and suffered more frequently from SUD than boys with ADHD [46,55]. In contrast, boys receiving pharmacological treatment for ADHD consumed less alcohol than those in the control group although this finding was not the same for adolescent girls with and without ADHD [53].	Adolescent boys and girls with more severe childhood ADHD initiated alcohol use earlier and drank alcohol more frequently, with the risk being similar for both sexes [45,59].
Marijuana and cannabis	Adolescent girls with ADHD had more problems with cannabis [43] and marijuana use than boys with ADHD [44,45].	Adolescents with more severe childhood ADHD initiated marijuana use earlier and used it more frequently, with the risk being similar for both sexes [45].
Other substances	Adolescent girls with ADHD suffered more SUD on other substances compared to boys with the same disorder [43] and girls without ADHD [59]. In turn, adolescent boys with SUD had more symptoms of ADHD and conduct disorder than female swith SUD [51].	

Note: ADHD, attention-deficit hyperactivity disorder; SUD, substance use disorder.

**Table 3:** Sex differences in the prevalence of attention-deficit hyperactivity disorder and pharmacological therapy.

	Girls/Boys	No Sex Differences
Prevalence	Girls were diagnosed with ADHD less frequently than boys (1 in 43 versus 1 in 10, respectively) [49] and presented fewer symptoms [49,54]. The prevalence of ADHD was lower in girls than in boys [41,50].	Hyperactive–impulsive type ADHD was most frequent and the combined type was the least frequent in both sexes [54].
Pharmacological therapy	Adolescent girls with ADHD were less likely to receive treatment (18.7%) than boys (28.4%) [40].	

Note: ADHD, attention-deficit hyperactivity disorder.

**Table 4:** Sex differences in cognitive and academic functioning.

	Girls/Boys	No Sex Differences
Cognitive functioning	Adolescent girls with ADHD scored higher for cognitive impulsivity and motor impulsivity [47]; they made made more errors of omission and had more deficits in design memory, general visual memory, verbal arithmetic skills, and working memory skills [56]; they also had higher processing and encoding speed scores, greater inhibitory control, and lower vocabulary scores than boys with ADHD [57]. Compared to boys, girls with ADHD had a more impaired neuropsychological profile in terms of executive functions [58].	
Academic functioning		ADHD was associated with poor academic performance (course repetitions, low grades, etc.) in both girls and boys [50]. No sex differences were found in any of the WISC-III subtests (block design, digits, symbol search, and arithmetic) [57].

Note: ADHD, attention-deficit hyperactivity disorder.

**Table 5:** Sex differences in other variables.

	Girls/Boys	No Sex Differences
Self-harm, suicidal ideation, and suicidal acts	ADHD was associated with a higher tendency towards suicidal acts, suicidal ideation, and self-harm in girls [48]. However, suicide attempts were significantly higher in boys with ADHD [50].	The results of the study by Kessler et al. (2014) [50] indicated that suicidal ideation and suicide planning occured at the same rate in adolescent boys and girls with ADHD.
Psychological problems		ADHD was associated with more psychological problems, distress, and stress in both sexes [50].
Motor function development	Both girls with ADHD the control group showed better motor development with age, while boys with ADHD showed minimal improvements [42].	
Obesity	Adolescent boys and girls with ADHD who received medication for the condition showed a lower prevalence of obesity but to a lesser extent in girls [41].	

Note: ADHD, attention-deficit hyperactivity disorder.

## Data Availability

Data from this study are available from the corresponding author upon request.
